# Supplementary material for: Domestication Gene Mlx and Its Partner Mondo Are Involved in Controlling the Larval Body Size and Cocoon Shell Weight of Bombyx mori
Source: Int J Mol Sci. 2024 Mar 18;25(6):3427. doi: 10.3390/ijms25063427 (PMC10970504; doi:10.3390/ijms25063427)
Supplement: Supplementary file 1 [file ijms-25-03427-s001.zip › ijms-2886521-supplementary.pdf]

>Bm1
TGAAGTCGTCGTGGCCTAAAGGATAAGACGTCCGGTGCATTCGTATCGAGCGATGCAC
TGGTGTTCGAATCTCGCTGGCGGGCACCAATTTTTCTAATGAAATACGTACCCAACAAA
TGTTCACGATTGGCCTCCACGATGAAGGAATAACATCGTGCAATATAAATCAAACCCGC
AAAATTATAATTTGCGTAATCACTGGTGGTAGGACCTCTTGTGAGTCCGCACGGGTAGG
TACCACCGCCCCGCCTATTTCTGCCGTGAAGCAGTAATGCGTTTCGGTTTGAAGGGTGG
GGCAGCCGTTGTGACTATACTGAGACCTTAGAACTATATCTCAAGGTGTGTGGCGCATT
TACGTTGTAGATGTCTATGGGCTCCAGTAACCACTTAACACCAGGTGGGCTGTGAGCTC
GTCCACATATCTAAGCAATAAAAAAAAAAAAAAAAAAAAAAAAATTT
Figure S1. The sequence of retrotransposon Bm1.

>DS1
TAAGCCCTTTCTAGGACACCCTTTATAATATATAATAATAAATAAATAAATATAACTAGCA
CTACTACAACATTAATTTATGCATAATTTTGAAGATTTTATAAGCTTGATTAAATTAAAAT
TACTCATATGTGTATTTTCTGTTGCTTGCAGAACTGGCATCATGAGCAGTCGATTGCCAT
ATTGAAAAAAGAAAATGTTGACGTTGACAGTCATCTGTGCTGTGACATAAATATTTTAA
ACGTTGACTTTTTGGCGGGAATGCGAGGAGTGAAGTTGTGTGATTTGATTCATTTTGTC
TATTTAGTGTTTCTTAAGATTTAAATGTGTAATAACGGTGGTTTATTAACTGTTCAATATC
TATGAAAGTGCACAAATGTGGGAAAACGAAACAAAGCCGCCGGACGCAACTTCTCGA
GATCCTCCAAAATGTCCACTGAAAAAATCTCAGTAAATGACCACCATTTTACTGAGATT
ATATTTCAACCCATATCATCTTATTTTATTTCATTTCATCCCAGTTGATTAACGTCTCAAAT
TAATCATCTTAATTTCATTTCACTCCATATTATCATTTTTCATAAAAATAAGAATATAAAGT
AAAGTAAGACATGACCTAAAGGTCTTAGTTACCAGGTCATAAAATCCCTTAAAAAAATA
ATTTTAAACGTAATTCTCGTACCTACCTATGTAGGTACTTATGTACGTAATTGTACCAAA
AGTCGGAAATAATACTGTATTTTAGTTATCAGTAATTTGAAATTTGATTTTAGTATTACGT
TACGTAAATGTTACGTAACCACATGGTTCAAAAGTGATTTAATTCGGGTCATGAAGTAC
TTGTCCAAGACTATCTTATATTTATAATGTTATCAATGTATTCTTCATACCCTACATAGAAA
TAATGGAACAAGGCACCATGAACTCCGAAAAGTGAAAGATCGCATTAATGTAATGTTTT
ATTTTATTAAAGTTTCATTATAACAATTAATATCCCAAT
>DS2
TAAGCCCTTTCTAGGACACCCTTTATAATATATAATAATAAATAAATAAATATAACTAGCA
CTACTACAACATTAATTTATGCATAATTTTGAAGATTTTATAAGCTTGATTAAATTAAAAT
TACTCATATGTGTATTTTCTGTTGCTTGCAGAACTGGCATCATGAGCAGTCGATTGCCAT
ATTGAAAAAAGAAAATGTTGACGTTGACAGTCATCTGTGCTGTGACATAAATATTTTAA
ACGTTGACTTTTTGGCGGGAATGCGAGGAGTGAAGTTGTGTGATTTGATTCATTTTGTC
TATTTAGTGTTTCTTAAGATTTAAATGTGTAATAACGGTGGTTTATTAACTGTTCAATATC
TATGAAAGTGCACAAATGTGGGAAAACGAAACAAAGCCGCCGGACGCAACTTCTCGA
GATCCTCCAAAATGTCCACTGAAAAAATCTCAGTAAATGACCACCATTTTACTGAGATT
ATATTTCAACCCATATCATCTTATTTTATTTCATTTCATCCCAGTTGATTAACGTCTCAAAT
TAATCATCTTAATTTCATTTCACTCCATATTATCATTTTTCATAAAAATAAGAATATAAAGT
AAAGTAAGACATGACCTAAAGGTCTTAGTTACCAGGTCATAAAATCCCTTAAAAAAATA
ATTTTAAACGTAATTCTCGTACCTACCTATGTAGGTACTTATGTACGTAATTGTACCAAA
AGTCGGAAATAATACTGTATTTTAGTTATCAGTAATTTGAAATTTGATTTTAGTATTACGT
TACGTAAATGTTACGTAACCACATGGTTCAAAAGTGATTTAATTCGGGTCATGAAGTAC

TTGTCCAAGACTATCTTATATTTATAATGTTATCAATGTATTCTTCATACCCTACATAGAAA
TAATGGAACAAGGCACCATGAACTCCGAAAAGTGAAAGATCGCATTAATGTAATGTTTT
ATTTTATTAAAGTTTCATTATAACAATTAATATCCCAAT
>DS3
TAAGCCCTTTCTAGGACACCCTTTATAATATATAATAATAAATAAATAAATATAACTAGCA
CTACTACAACATTAATTTATGCATAATTTTGAAGATTTTATAAGCTTGATTAAATTAAAAT
TACTCATATGTGTATTTTCTGTTGCTTGCAGAACTGGCATCATGAGCAGTCGATTGCCAT
ATTGAAAAAAGAAAATGTTGACGTTGACAGTCATCTGTGCTGTGACATAAATATTTTAA
ACGTTGACTTTTTGGCGGGAATGCGAGGAGTGAAGTTGTGTGATTTGATTCATTTTGTC
TATTTAGTGTTTCTTAAGATTTAAATGTGTAATAACGGTGGTTTATTAACTGTTCAATATC
TATGAAAGTGCACAAATGTGGGAAAACGAAACAAAGCCGCCGGACGCAACTTCTCGA
GATCCTCCAAAATGTCCACTGAAAAAATCTCAGTAAATGACCACCATTTTACTGAGATT
ATATTTCAACCCATATCATCTTATTTTATTTCATTTCATCCCAGTTGATTAACGTCTCAAAT
TAATCATCTTAATTTCATTTCACTCCATATTATCATTTTTCATAAAAATAAGAATATAAAGT
AAAGTAAGACATGACCTAAAGGTCTTAGTTACCAGGTCATAAAATCCCTTAAAAAAATA
ATTTTAAACGTAATTCTCGTACCTACCTATGTAGGTACTTATGTACGTAATTGTACCAAA
AGTCGGAAATAATACTGTATTTTAGTTATCAGTAATTTGAAATTTGATTTTAGTATTACGT
TACGTAAATGTTACGTAACCACATGGTTCAAAAGTGATTTAATTCGGGTCATGAAGTAC
TTGTCCAAGACTATCTTATATTTATAATGTTATCAATGTATTCTTCATACCCTACATAGAAA
TAATGGAACAAGGCACCATGAACTCCGAAAAGTGAAAGATCGCATTAATGTAATGTTTT
ATTTTATTAAAGTTTCATTATAACAATTAATATCCCAAT
>DS4
TAAGCCCTTTCTAGGACACCCTTTATAATATATAATAATAAATAAATAAATATAACTAGCA
CTACTACAACATTAATTTATGCATAATTTTGAAGATTTTATAAGCTTGATTAAATTAAAAT
TACTCATATGTGTATTTTCTGTTGCTTGCAGAACTGGCATCATGAGCAGTCGATTGCCAT
ATTGAAAAAAGAAAATGTTGACGTTGACAGTCATCTGTGCTGTGACATAAATATTTTAA
ACGTTGACTTTTTGGCGGGAATGCGAGGAGTGAAGTTGTGTGATTTGATTCATTTTGTC
TATTTAGTGTTTCTTAAGATTTAAATGTGTAATAACGGTGGTTTATTAACTGTTCAATATC
TATGAAAGTGCACAAATGTGGGAAAACGAAACAAAGCCGCCGGACGCAACTTCTCGA
GATCCTCCAAAATGTCCACTGAAAAAATCTCAGTAAATGACCACCATTTTACTGAGATT
ATATTTCAACCCATATCATCTTATTTTATTTCATTTCATCCCAGTTGATTAACGTCTCAAAT
TAATCATCTTAATTTCATTTCACTCCATATTATCATTTTTCATAAAAATAAGAATATAAAGT
AAAGTAAGACATGACCTAAAGGTCTTAGTTACCAGGTCATAAAATCCCTTAAAAAAATA
ATTTTAAACGTAATTCTCGTACCTACCTATGTAGGTACTTATGTACGTAATTGTACCAAA
AGTCGGAAATAATACTGTATTTTAGTTATCAGTAATTTGAAATTTGATTTTAGTATTACGT
TACGTAAATGTTACGTAACCACATGGTTCAAAAGTGATTTAATTCGGGTCATGAAGTAC
TTGTCCAAGACTATCTTATATTTATAATGTTATCAATGTATTCTTCATACCCTACATAGAAA
TAATGGAACAAGGCACCATGAACTCCGAAAAGTGAAAGATCGCATTAATGTAATGTTTT
ATTTTATTAAAGTTTCATTATAACAATTAATATCCCAAT
>WS1-Short
TAAGCCCTTTCTAGGACACCCTTTATAAAATAAATATAACTAGCACTACTACAACATTAA
TATAAGCATACTTCTGAAGATTTTATAAGCTTGATTAAATTAAAATTACTTAAATTCTATT
TAATAAATGTGTATTTTCTGTTGCTTGCAGAACTGGCATCATGAGCAGTCGATTGCCATA
TTGAAAAAAGAAAATGTTGACGTTGACAGTCATCTGTGCTGTGACATAAATATTATAAA

CGTTGACTTTTTGGCGGGAATGCGAGGAGTGAAGTTGTGTGATTTGATTCATTTTGTCT
ATTTAGTGTTTCTTAAGATTTAAATGTGTAATAACGGTGGTTTATTAACTGTTTAATATCT
ATGAAAGTGCACAAATGTGAGAAAACGAAACAAAGCCGCCGGACGCAACTTCACGG
GATCCTCCAAAATGTCCACTGAAAAAATCTCAGTAAATGACCACCATTTTACTGAGATT
ATATTTCAACCCATATCATCTCATCTCATTTTATTTCATTTCATCCCAGTTGATTAACGTCT
CAAATTAATCATCTTCATTTCATTTCACTCCATATTATCATTTTCATAAAAATAAGAACAT
AAAGTAAAGTAAGACATGACCTAAAGGTCTTAGTTACCAGGTCATAAAATCCCTTAAA
AAAATTATTTAAACGTAATTCTCGTACCTACCTACGTAATATATGTAGGTACGTAATTGT
ACCAAAAGTCGGAAATAATACTGTTATTTTAGTTATCAGTAATTTGAAATTTGATTTTAG
TATTACGTTACGTAAATGTTACGTAATCATATGGTTCAAAAGTGATTTAATTCGGGTCAT
GAAGTACTTGTCCAAGACTATCTTATATTTATAATGTTATCAATGTATTCTTCATACCCTAC
ATAGAAATAATGGAACAAGGCACCATGAACTCCGAAAAGTGAAAGATCGCATTAATGT
AATGTTTTATTCTATTAAAGTTTGATTATAACAATTAATATCCCAAT
>WS2-Short
TAAGCCCTTTCTAGGACACCCTTTATACTATATAATAATAAATAAATAAATATAACTAGCA
CTACTACAACATTAATTTATGCATACTTTTGAAGATTTTATAAGCTTGATTAAATTAAAAT
TATTTATATGTGTATTTTCTGTTGCTTGCAGAACTGGCATCATGAGCAGTCGATTGCCATA
TTGAAAAAAGAAAATGTTGACGTTGACAGTCATCTGTGCTGTGACATAAATATTTTAAA
CGTTGACTTTTTGGCGGGAATGCGAGGAGTGAAGTTGTGTGATTTGATTCATTTTGTCT
ATTTAGTGTTTCTTAAGGTTTAAATGTGTAATAACGGTGGTTTATTAACTGTTCAATATCT
ATGAAAGTGCACAAATGTGGGAAAACGAAACAAAGCCGCCGGACGCAACTTCTCGGG
ATCCTCCAAAATGTCCACTGAAAAAATCTCAGTAAATGACCACCATTTTACTGAGATTA
TATTTCAACCCATATCATCTTATTTTATTTCATTTCATCCCAGTTGATTAACGTCTCAAATT
AATCATCTTCATTTCATTTCACTCCATATTATCATTTTTCATAAAAATAAGAACATAAAGT
AAAGTAAGACATGACCTAAAGGTCTTAGTTACCAGGTCATAAAATCCCTTAAAAAAATA
ATTTTAAACGTAATTCTCGTATATACCTACCTATGTAGGTACTTATGTACGTAATTGTACC
AAAAGTCGGAAATAATACTGTTATTTTAGTTATCAGTAATTTGAAATTTGATTTTAGTATT
ACGTTACGTAAATGTTACGTAACCACATGGTTCAAAAGTGATTTAATTCGGGTCATGAA
GTACTTGTCCAAGACTATCTTATATTTATAATGTTATCAATGTATTCTTCATACCCTACATA
GAAATAATGTAACAAGGCACCATGAACTCCGAAAAGTGAAAGATCGCATTAATGTAAT
GTTTTATTTTATTAAAGTTTGATTATAACAATTAATATCCCAAT
>WS3-Short
TAAGCCCTTTCTAGGACACCCTTTATAAAATAAATATAACTATAACTACTACAACATTAAT
TTATGCATACTTCTGAAGATTTTATAAGCTTGATTAAATTAAAATTACTTAAATTCTATTTA
TTAAATGTGTATTTTCTGTTGCTTGCAGAACTGGCATCATGAGCAGTCGATTGCCATATT
GAAAAAAGAAAATGTTGACGTTGACAGTCATCTGTGCTGTGGCATAAATATTATAAACG
TTGACTTTTTGGCGGGAATGCGAGGAGTGAAGTTGTGTGATTTGATTCATTTTGTCTATT
TAGTGTTTCTTAAGATTTAAATGTGTAATAACGGTGGTTTATTAACTGTTTAATATCTATG
AAAGTGAACAAATGTGACGCAACTTCTCGGGATCCTCCAAAATGTTCACTGAAAAAAT
CTCAGTAAATGACCACCATTTTACTGAGATTATATTTCAACCCATATCATCCCATCTCATT
TTATTTCATTTCATCCCAGTTGATCAACGTCTCAAATTAATCATCTTCATTTCACTCCATA
TTATCATTTTTCATAAGAATAAGAACATAAAGTAAAGTAAGACATGACCTAAAGGTCTT
AGTTACCAGGTCATAAAATCCCTTAAAAAATTATTTTAAACGTAATTCTCTTACCTACCT
ACGTAATTTATGTAGGTACGTAATTGTACCAAAAGTCGGAAATAATACTGTTATTTTAGT

TATCAGTAATTTGAAATTTGATTTTAGTATTACGTTACGTAAATGTTACGTAACCATATGG
TTCAAAAGTGATTTAATTCGGGTCATGAAGTACTTGTCCAAGACTATCTTATATTTATAAT
GTTATCAATGTATTCTTCATACCCTACATAGAAATAATGGAACAAGGCACCATGAACTCC
GAAAAGTGAAAGATCGCATTAATGTAATGTTTTATTTTATTAAA
GTTTCATTATAACAATTAATATCCCAAT
>WS4-Short
TAAGCCCTTTCTAGGACACCCTTTATACTATATAATAATAAATAAATAAATATAACTAGCA
CTACTACAACATTAATTTATGCATACTTTTGAAGATTTTATAAGCTTGATTAAATTAAAAT
TACTTATATGTGTATTTTCTGTTGCTTGCAGAACTGGCATCATGAGCAGTCGATTGCCAT
ATTGAAAAAAGAAAATGTTGACGTTGACAGTCATCTGTGCTGTGACATAAATATTTTAA
ACGTTGACTTTTTGGCGGGAATGCGAGGAGTGAAGTTGTGTGATTTGATTCATTTTGTC
TATTTAGTGTTTCTTAAGGTTTAAATGTGTAATAACGGTGGTTTATTAACTGTTCAATATC
TATGAAAGTGCACAAATGTGGGAAAACGAAACAAAGCCGCCGGACGCAACTTCTCGG
GATCCTCCAAAATGTCCACTGAAAAAATCTCAGTAAATGACCACCATTTTACTGAGATT
ATATTTCAACCCATATCATCTTATTTTATTTCATTTCATCCCAGTTGATTAACGTCTCAAAT
TAATCATCTTCATTTCATTTCACCCCATATTATCATTTTCCATAAAAATAAGAACATAAAG
TAAAGTAAGACATGACCTAAAGGTCTTAGTTACCAGGTCATAAAATCCCTTAAAAAAAT
AATTTTAAACGTAATTCTCGTACCTACCTATGTAGGTACTTATGTACGTAATTGTACCAA
AAGTCGGAAATAATACTGTTATTTTAGTTATCAGTAATTTGAAATTTGATTTTAGTATTAC
GTTACGTAAATGTTACGTAACCACATGGTTCAAGAGTGATTTAATTCGGGTCATGAAGT
ACTTGTCCAAGACTATCTTATATTTATAATGTTATCAATGTATTCTTCATACCCTACATAGA
AATAATGGAACAAGGCACCATGAACTCCGAAAAGTGAAAGATCGCATTAATGTAATGT
TTTATTTTATTAAAGTTTCATTATAACAATTAATATCCCAAT
>WS1-Long
TAAGCCCTTTCTAGGACACCCTTTATAATATATAATAATAAATAAATAAATATAACTAGCA
CTACTACAACATTAATTTATGCATACTTTTGAAAATTTTATAAGCTTGATTAAATTAAAAT
TACTTAAATATGTATTTTCTGTTGCTTGCAGAACTGGCATCATGGGCAGTCGATTGCCAT
ATTGAAAAAAGAAAATGTTGACGTTGACAGTCATCTGTGCTGTGACATAAATATTTTAA
ACGTTGACTTTTTGGCGGGAATGCGAGGAGTGAAATTGTGTGATTTTGAAGTCGTCGT
GGCCTAAAGGATAAGACGTCCGGTGCATTCGTATCGAGCGATGCACTGGTGTTCGAATC
TCGCTGGCGGGCACCAATTTTTCTAATGAAGTACGTACCCAACAAATGTTCACGATTGG
CCTCCACGATGAAGGAATAACATCGTGCAATATAAATCAAACCCGCAAAATTATAATTT
GCGTAATCACTGGTGGTAGGACCTCTTGTGAGTCCGCACGGGTAGGTACCACCGCCCC
GCCTATTTCTGCCGTGAAGCAGTAATGCGTTTCGGTTTGAAGGGTGGGGCAGCCGTTG
TGACTATACTGAGACCTTAGAACTATATCTCAAGGTGTGTGGCGCATTTACGTTGTAGAT
GTCTATGGGCTCCAGTAACCACTTAACACCAGGTGGACTGTGAGCTCGTCCACATATCT
AAGCAATAAAAAAAAAAAAAAAAAAAAATTTGATTCATTTTGTCTATTTAGTGTTTCTTA
AGGTTTAAATGTGTAATAACGGTGGTTTATTAACTGTTCAATATCTATGAAAGTGCACAA
ATGTGGGAAAACGAAACAAAGCCGCCGGACGCAACTTCTCGGGATCCTCCAAAATGT
CCACTGAAAAAATCTCAGTAAATGACCACCATTTTACTGAGATTATATTTCAACCCATAT
CATCTTATTTTATTTCATTTCATCCCAGTTGATTAACGTCTCAAATTAATCATCTTCATTTC
ATTTCACTCCATATTATCATTTTTCATAAAAATAAGAACATAAAGTAAAGTAAGACATGA
CCTAAAGGTCTTAGTTACCAGGTCATAAAATCCCTTAAAAAAATTATTTTAAACGTAATT
CTCGTACCTACCTATGTAGGTACTTATGTACGTAATTGTACCAAAAGTCGGAAATAATAC

TGTTATTTTAGTTATCAGTAATTTGAAATTTGATTTTAGTATTACGTTACGTAAATGTTAC
GTAACCACATGGTTCAAAAGTGATTTAATTCGGGTCATGAAGTACTTGTCCAAGACTAT
CTTATATTTATAATGTTATCAATGTATTCTTCATACCCTACATAGAAATAATGGAACAAGG
CACCATGTACTCCGAAAAGTGAAAAATCGCATTAATGTAATGTTTTATTTTATTAAAGTT
TGATTATAACAATTAATATCCCAAT
>WS2-Long
TAAGCCCTTTCTAGGACACCCTTTATAATATATAATAATAAATAAATAAATATAACTAGCA
CTACTACAACATTAATTTATGCATACTTTTGAAAATTTTATAAGCTTGATTAAATTAAAAT
TACTTAAATATGTATTTTCTGTTGCTTGCAGAACTGGCATCATGAGCAGTCGATTGCCAT
ATTGAAAAAAGAAAATGTTGACGTTGACAGTCATCTGTGCTGTGACATAAATATTTTAA
ACGTTGACTTTTTGGCGGGAATGCGAGGAGTGAAATTGTGTGATTTTGAAGTCGTCGT
GGCCTAAAGGATAAGACGTCCGGTGCATTCGTATCGAGCGATGCACTGGTGTTCGAATC
TCGCTGGCGGGCACCAATTTTTCTAATGAAATACGTACCCAACAAATGTTCACGATTGG
CCTCCACGATGAAGGAATAACATCGTGCAATATAAATCAAACCCGCAAAATTATAATTT
GCGTAATCACTGGTGGTAGGACCTCTTGTGAGTCCGCACGGGTAGGTACCACCGCCCC
GCCTATTTCTGCCGTGAAGCAGTAATGCGTTTCGGTTTGAAGGGTGGGGCAGCCGTTG
TGACTATACTGAGACCTTAGAACTATATCTCAAGGTGTGTGGCGCATTTACGTTGTAGAT
GTCTATGGGCTCCAGTAACCACTTAACACCAGGTGGGCTGTGAGCTCGTCCACATATCT
AAGCAATAAAAAAAAAAAAAAAAAAAAAAAATTTGATTCATTTTGTCTATTTAGTGTTTC
TTAAGGTTTAAATGTGTAATAACGGTGGTTTATTAACTGTTCAATATCTATGAAAGTGCA
CAAATGTGGGAAAACGAAACAAAGCCGCCGGACGCAACTTCTCGGGATCCTCCAAAA
TGTCCACTGAAAAAATCTCAGTAAATGACCACCATTTTACTGAGATTATATTTCAACCC
ATATCATCTTATTTTATTTCATTTCATCCCAGTTGATTAACGTCTCAAATTAATCATCTTCA
TTTCATTTCACTCCATATTATCATTTTTCATAAAAATAAGAACATAAAGTAAAGTAAGAC
ATGACCTAAAGGTCTTAGTTACCAGGTCATAAAATCCCTTAAAAAAATTATTTTAAACGT
AATTCTCGTACCTACCTATGTAGGTACTTATGTACGTAATTGTACCAAAAGTCGGAAATA
ATACTGTTATTTTAGTTATCAGTAATTTGAAATTTGATTTTAGTATTACGTTACGTAAATGT
TACGTAACCACATGGTTCAAAAGTGATTTAATTCGGGTCATGAAGTACTTGTCCAAGAC
TATCTTATATTTATAATGTTATCAATGTATTCTTCATACCCTACATAGAAATAATGGAACAA
GGCACCATGAACTCCGAAAAGTGAAAAATCGCATTAATGTAATGTTTTATTTTATTAAA
GTTTGATTATAACAATTAATATCCCAAT
>WS3-Long
TAAGCCCTTTCTAGGACACCCTTTATAATATAAATATAACTAGCACTACTACAACATTAAT
TTATGCATACTTCTGAAGATTTTAGAAGCTTGATTAAATTAAAATTACTTAAATTCTATTT
ATTAAATGTGTATTTTCTGTTGCTTGCAGAACTGGCATCATGAGTAGTCGATTGCCATAT
TGAAAAAAGAAAATGTTGACGTTGACAGTCATCTGTGCTGTGACATCAATATTATAAAC
GTTGGAGGACTTTTTGGCGGGAACGCGAGGAGTGAAGTTGTGTGATTTGTTTTATTTTG
TCCATTTAGTGTTTCTTCAGGTTTAAATGTGTAATAACGGTGGTTTATTAACTATTTAATA
TCTGTGAAAGTGCACAAATGTGGGAAAATGAAACAAAGCCGCTGGACGCAACTTCTC
GGGATCCTCCAAAAAGTCCACTGAAAAAATCTTAGTAAATGACCACCGTTTTACTAAG
ATTATATTTCATTCCATATCATCTCATTTCATTTAATTTCATCCCAGTTGATTAACGTCTCA
AATTAATCATTTTCATTTCATTTCACTCCATATTATCATTTTTCATAAAAATAAGAATATAA
ACTAAAATAAGACATGACTTAAAGGTCTTAGTTACCAGGTCATAAAATCACTTAAAAAA
AAAATTATAAACGTTGACTTTTTGGCGGGAATGCGAGGAGTGGAGTTGTGTGATTTGAT

TCATTTTGTCTATTTAGTGTTTCTTAAGATTTAAATGTGTAATAACGGTGGTTTATTAACT
GTTTAATATCTATGAAAGTGCACAAATGTGAGAAAACGAAACAAAGCCGCCGGACGCA
ACTTCTCGGGATCCTCCAAAGTGTCCACTGAAAAAATCTCAGTAAATGACCACCATTTT
ACTGAGATTATATTTCAACCCATATCATCTCATCTCATTTTATTTCATTTCATCCCAGTTGA
TTATCGTCTCAAATTAATCATCTTCATTTCATTTCACTCCATATTATCATTTTTCATAAAAA
TAAGAACATAAAGTAAAGTAAGACATGACCTAAAGGTCTTAGTTACCAGGTCATAAAAT
CCCTTAAAAAAATTATTTTAAACGTAATTCCCGTATACCTACGTAATTGTACCAAAAGTC
GGAAATAATACTGTTATTTTAGTTATCAGTAATTTGAAATTTGATTTTAGTATTACGTTAC
GTAAATGTTACGTAACCATATGGTTCAAAAGTGATTTAATTCGGGTCATGAAGTACTTGT
CCAAGACTATCTTATATTTATAATGTTATCAATGTATTCTTCATACCCTACATAGAAATAAT
GGAACAAGGCACCATGAACTCCGAAAAGTGAAAGATCGCATTAATGTAATGTTTTATTT
TATTAAAGTTTGATTATAACAATTAATATCCCAAT

>WS4-Long

TAAGCCCTTTCTAGGACACCCTTTATAATATAAATATAACTAGCACTACTACAACATTAAT
TTATGCATACTTCTGAAGATTTTAGAAGCTTGATTAAATTAAAATTACTTAAATTCTATTT
ATTAAATGTGTATTTTCTGTTGCTTGCAGAACTGGCATCATGAGTAGTCGATTGCCATAT
TGAAAAAAGAAAATGTTGACGTTGACAGTCATCTGTGCTGTGACATCAATATTATAAAC
GTTGGAGGACTTTTTGGCGGGAACGCGAGGAGTGAAGTTGTGTGATTTGTTTTATTTTG
TCCATTTAGTGTTTCTTCAGGTTTAAATGTGTAATAACGGTGGTTTATTAACTATTTAATA
TCTGTGAAAGTGCACAAATGTGGGAAAATGAAACAAAGCCGCTGGACGCAACTTCTC
GGGATCCTCCAAAAAGTCCACTGAAAAAATCTTAGTAAATGACCACCGTTTTACTAAG
ATTATATTTCATTCCATATCATCTCATTTCATTTAATTTCATCCCAGTTGATTAACGTCTCA
AATTAATCATTTTCATTTCACTTCACTCCATATTATCATTTTTCATAAAAATAAGAATATAA
ACTAAAATAAGACATGACTTAAAGGTCTTAGTTACCAGGTCATAAAATCACTTAAAAAA
AAAATTATAAACGTTGACTTTTTGGCGGGAATGCGAGGAGTGAAGTTGTGTGATTTGAT
TCATTTTGTCTATTTAGTGTTTCTTAAGATTTAAATGTGTAATAACGGTGGTTTATTAACT
GTTTAATATCTATGAAAGTGCACAAATGTGAGAAAACGAAACAAAGCCGCCGGACGCA
ACTTCTCGGGATCCTCCAAAATGTCCACTGAAAAAATCTCAGTAAATGACCACCATTTT
ACTGAGATTATATTTCAACCCATATCATCTCATCTCATTTTATTTCATTTCATCCCAGTTGA
TTATCGTCTCAAATTAATCATCTTCATTTCATTTCACTCCATATTATCATTTTTCATAAAAA
TAAGAACATAAAGTAAAGTAAGACATGACCTAAAGGTCTTAGTTACCAGGTCATAAAAT
CCCTTAAAAAAATTATTTTAAACGTAATTCCCGTATACCTACGTAATTGTACCAAAAGTC
GGAAATAATACTGTTATTTTAGTTATCAGTAATTTGAAATTTGATTTTAGTATTACGTTAC
GTAAATGTTACGTAACCATATGGTTCAAAAGTGATTTAATTCGGGTCATGAAGTACTTGT
CCAAGACTATCTTATATTTATAATGTTATCAATGTATTCTTCATACCCTACATAGAAATAAT
GGAACAAGGCACCATGAACTCCGAAAAGTGAAAGATCGCATTAATGTAATGTTTTATTT
TATTAAAGTTTGATTATAACAATTAATATCCCAAT

Figure S2. The amplified sequences of the upstream region of *Mlx* translation start codon in domesticated and wild silkworm. Domesticated silkworm: DS1-4, wild silkworm: WS1-2.

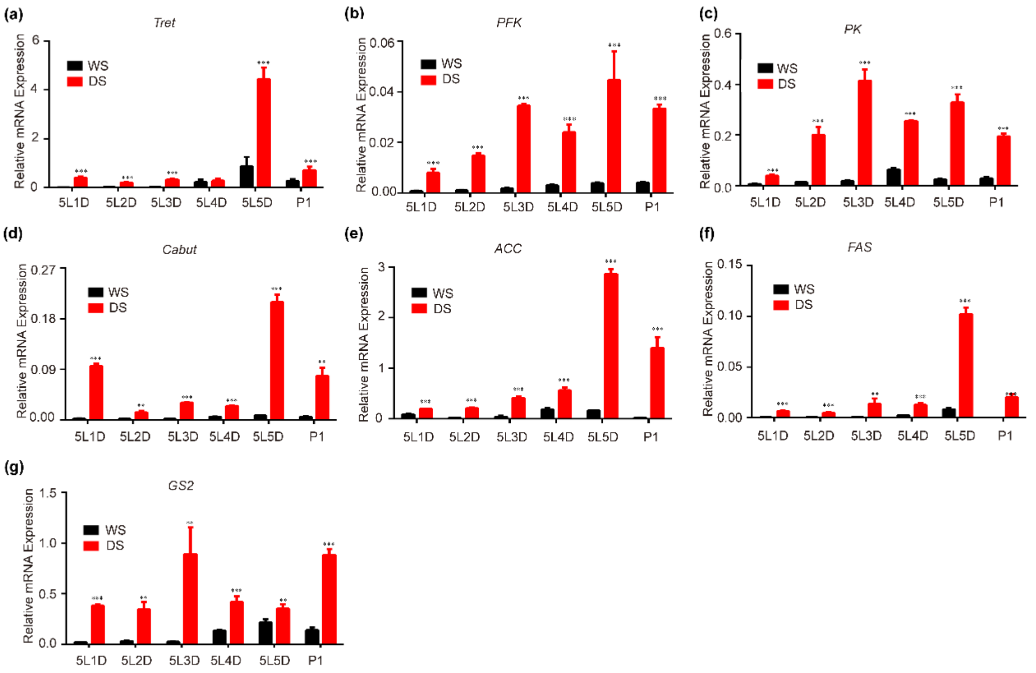

Figure S3. The expression of some metabolism-related genes and factors in domesticated and wild silkworm. (a-g) Comparing the expression of *Tret*, *PFK*, *PK*, *Cabut*, *ACC*, *FAS*, and *GS2* in the 5th instar and pupal stage of the fat body in domesticated and wild silkworms (DS and WS). 5L1D: the 1st day of the 5th instar, 5L2D: the 2nd day of the 5th instar, 5L3D: the 3rd day of the 5th instar, 5L4D: the 4th day of the 5th instar, 5L5D: the 5th day of the 5th instar, P1: the 1st day of pupal stage. Error bars show the SD. Significant differences: $^{**}p < 0.01$, $^{***}p < 0.001$.

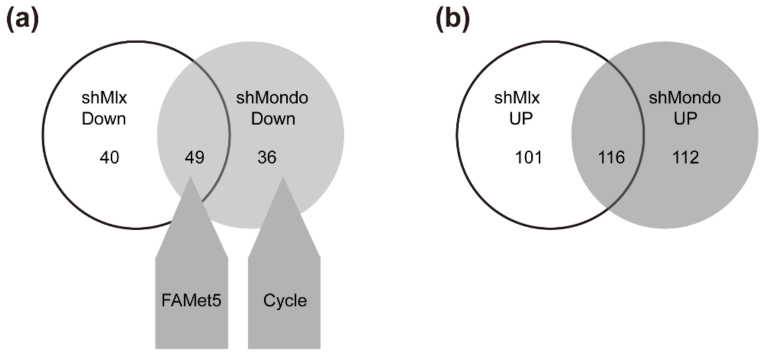

Figure S4. The Venn diagram of transcriptomes with *Mlx* or *Mondo* knocked down by shRNA in BmNs cell. (a) The number of genes with decreased expression after interfering with *Mlx* or *Mondo*. (b) The number of genes with increased expression after interfering with *Mlx* or *Mondo*.

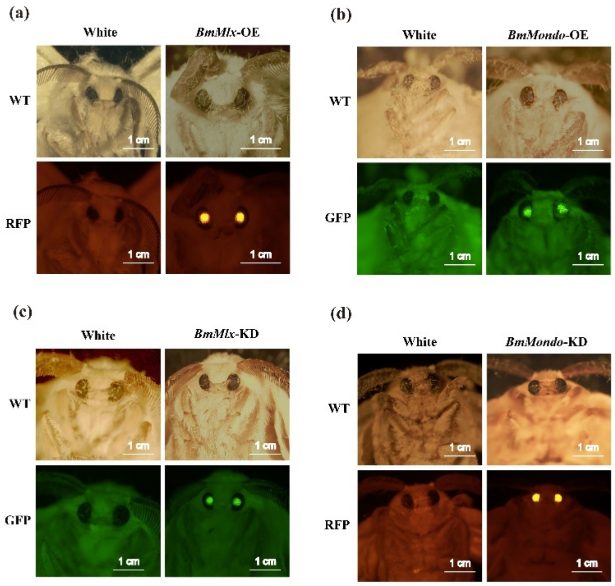

Figure S5. *Mlx* and *Mondo* transgenic strains were screened by fluorescence microscopy. (a): The transgenic overexpression of *Mlx* strain (*Mlx*-OE). (b): The transgenic overexpression of *Mondo* strain (*Mondo*-OE). (c): The transgenic knock down of *Mlx* strain (*Mlx*-KD). (d): The transgenic knock down of *Mondo* strain (*Mondo*-KD).

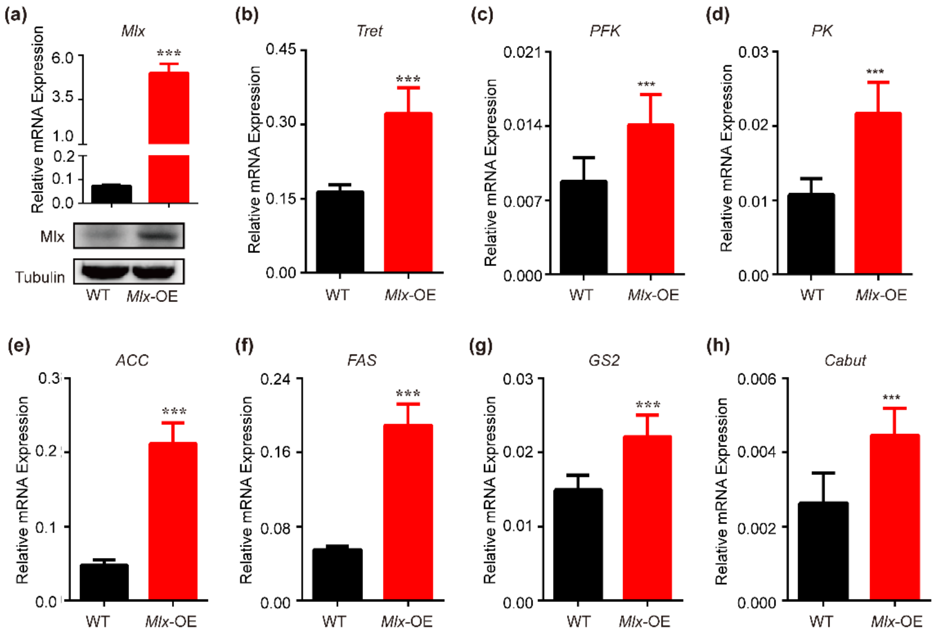

Figure S6. The expression of *Mlx* and target genes in transgenic *Mlx*-OE and WT samples. (a–h) The expression of *Mlx*, *Tret*, *PFK*, *PK*, *ACC*, *FAS*, *GS2*, and *Cabut* increased in the *Mlx*-OE strain. Error bars show the SD. Significant differences: ***$p < 0.001$.

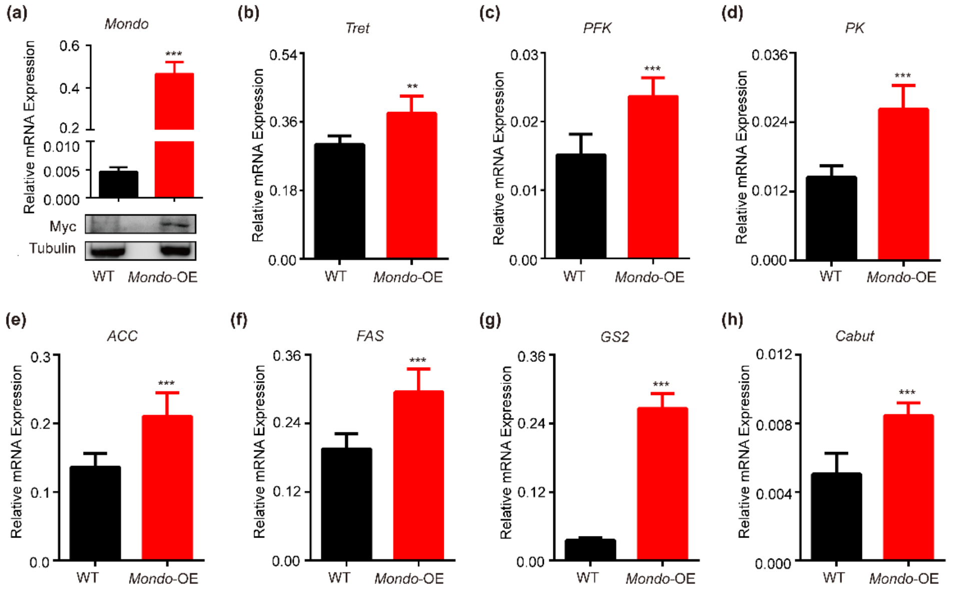

Figure S7. The expression of *Mondo* and target genes in transgenic *Mondo*-EO and WT samples. (a–h) The expression of *Mlx*, *Tret*, *PFK*, *PK*, *ACC*, *FAS*, *GS2*, and *Cabu*t increased in the *Mondo* -OE strain. Error bars show the SD. Significant differences: **p < 0.01, ***p < 0.001.

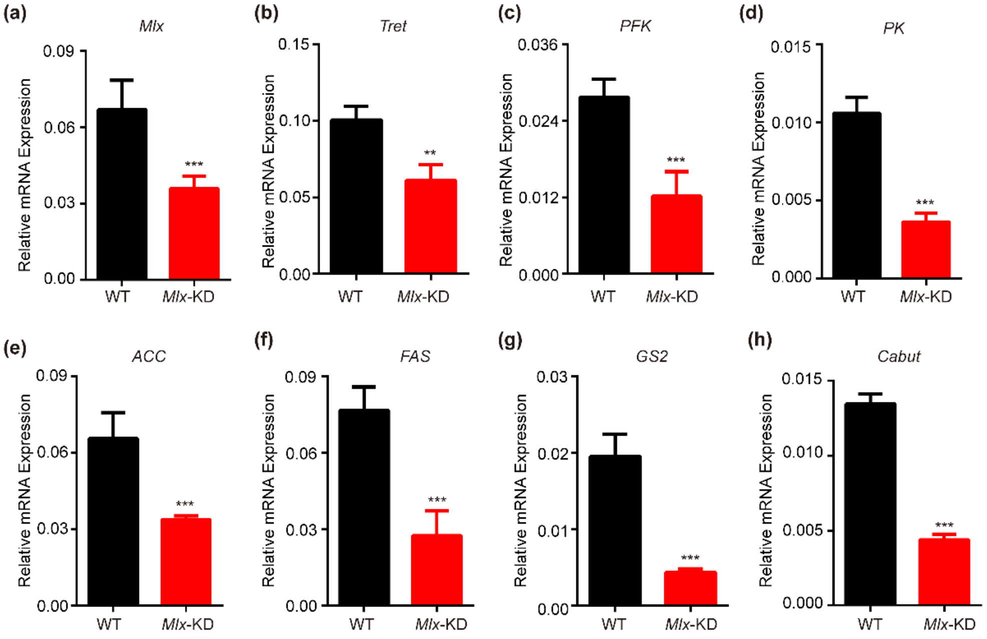

Figure S8. The expression of *Mlx* and target genes in transgenic *Mlx*-KD and WT samples. (a–h) The expression of *Mlx*, *Tret*, *PFK*, *PK*, *ACC*, *FAS*, *GS2*, and *Cabu*t decreased in the *Mlx*-OE strain. Error bars show the SD. Significant differences: **p < 0.01, ***p < 0.001.

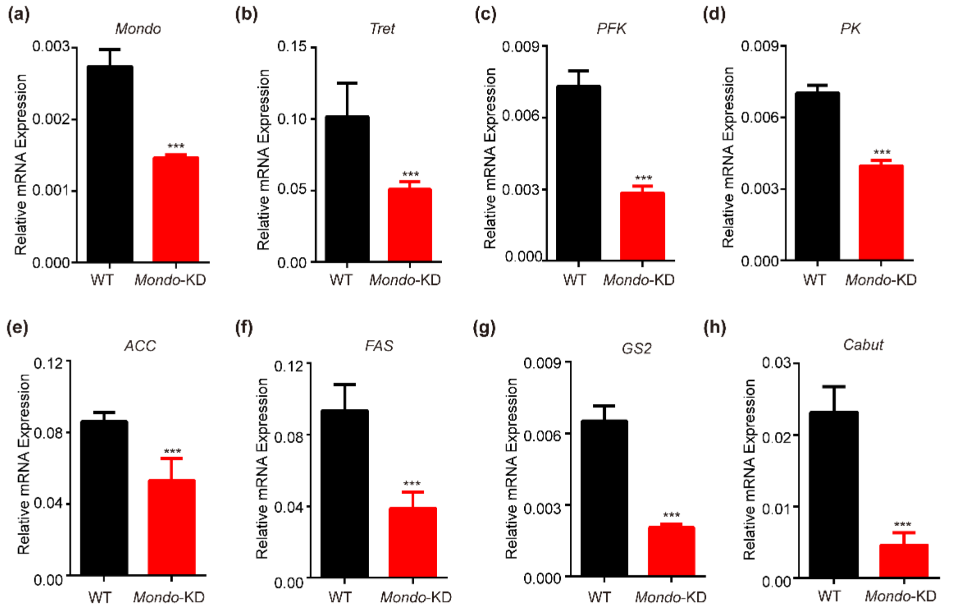

Figure S9. The expression of *Mondo* and target genes in transgenic *Mondo*-KD and WT samples. (a–h) The expression of *Mlx*, *Tret*, *PFK*, *PK*, *ACC*, *FAS*, *GS2*, and *Cabu*t decreased in the *Mondo*-OE strain. Error bars show the SD. Significant differences: ***p < 0.001.

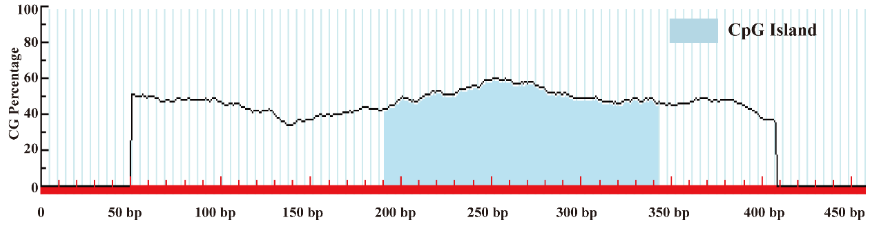

Figure S10. The CG percentage of Bm1 sequence.

Table S1. Domesticated and wild silkworm samples used in this study.

Species	Sample ID	Strain name	Location	TE
B. mori	DS2	872	China	-
B. mori	DS3	Qiubai	China	-
B. mori	DS4	Haoyue	China	-
B. mori	DS1	Dazao	China	-
B. mori	DS5	C108T	Japan	-
B. mori	DS6	N4	Japan	-
B. mori	DS7	b20	Japan	-
B. mori	DS8	c10	Japan	-
B. mori	DS9	c51	Japan	-
B. mori	DS10	d18	Japan	-
B. mori	DS11	e10	Japan	-
B. mori	DS12	f35	Japan	-
B. mori	DS13	g53	Japan	-
B. mori	DS14	k25	Japan	-

Species	Sample ID	Strain name	Location	TE
B. mori	DS15	n16	Japan	-
B. mori	DS16	o55	Japan	-
B. mori	DS17	o56	Japan	-
B. mori	DS18	p20	Japan	-
B. mori	DS19	p21	Japan	-
B. mori	DS20	p22	Japan	-
B. mori	DS21	p44	Japan	-
B. mandarina	WS1	Beibei	China	+
B. mandarina	WS2	Changshou	China	+
B. mandarina	WS3	Jiangsu	China	+
B. mandarina	WS4	Rongchang	China	+

Gray background represents the samples used to perform the polymorphism analysis.

Table S2. Potential binding transcription factors of Bm1.

Matrix Family	Detailed Family Information	Matrix Similarity	Sequence
SP1F	GC-Box factors SP1/GC	0.937	aggcgGGGCggtggtac
KLFS	Krueppel like transcription factors	0.891	ataggcGGGGcggtggtac
GLIF	GLI zinc finger family	0.938	accaccgCCCCgcctat
E2FF	E2F-myc activator/cell cycle regulator	0.973	aataGGCGgggcggtgg
KLFS	Krueppel like transcription factors	0.937	gaaataggcGGGGcggtgg
SP1F	GC-Box factors SP1/GC	0.903	gaaataGGCGgggcggt
HOXF	Hox clusters A, B, C, D	0.823	agcagTAATgcgtttcggt
HOMF	Homeodomain transcription factors	0.851	tcaaaccgAAACgcattac
IRFF	Interferon regulatory factors	0.945	ccacccttcaaaccGAAAcgcatta
CHRF	Cell cycle homology element	0.954	cggtTTGAagggt
ZF02	C2H2 zinc finger transcription factors 2	1	ggctgCCCCacccttcaaaccga
SAL2	Spalt-like transcription factor 2	0.953	aaggGTGGggc
SP1F	GC-Box factors SP1/GC	0.914	ttgaagGGTGgggcagc
KLFS	Krueppel like transcription factors	0.996	ttgaaGGGTggggcagccg

Table S3. Transcriptome of control vs. the knock down of *Mlx* in BmNs cells.

Up

ID	AveExpr	Foldchange	P.value
BGIBMGA000116	19.624103	3.2304346	0.0025358
BGIBMGA000117	195.16167	2.5595289	5.545E-16
BGIBMGA000135	62.392665	2.1423068	7.864E-05
BGIBMGA000196	19.698035	2.0522563	0.0486403
BGIBMGA000223	16.422248	2.3748691	0.0265613
BGIBMGA000395	21222.974	2.1502583	1.501E-14
BGIBMGA000458	20.255612	2.0220379	0.0431847
BGIBMGA000476	514.8335	2.3733773	1.029E-31
BGIBMGA000551	18.532224	2.3716452	0.0334874
BGIBMGA000561	13.044966	2.52876	0.0496366

ID	AveExpr	Foldchange	P.value
BGIBMGA000590	543.86276	2.7499058	1.055E-44
BGIBMGA000648	199.84405	2.0791779	1.419E-06
BGIBMGA000667	1905.3998	2.2741547	4.939E-85
BGIBMGA000691	24.248299	2.2361649	0.0160307
BGIBMGA000736	21.816913	2.9051963	0.0029713
BGIBMGA000876	37.448152	2.3237055	0.0013685
BGIBMGA001367	275.47526	3.3965357	3.852E-32
BGIBMGA001376	18.043965	4.8274793	0.00032
BGIBMGA001384	6.4310965	10.05204	0.009529
BGIBMGA001399	316.47885	2.0546119	1.62E-16
BGIBMGA001613	642.00881	2.1800302	7.577E-21
BGIBMGA001755	949.82259	2.2187386	1.039E-47
BGIBMGA001815	13.493701	3.1245851	0.0141404
BGIBMGA001836	22.70069	2.3163796	0.0114229
BGIBMGA001862	21.99046	2.5557269	0.006298
BGIBMGA001905	12.263037	3.980299	0.0065645
BGIBMGA001941	6.4495794	5.3384711	0.0359889
BGIBMGA002000	20.024059	2.1982355	0.0282093
BGIBMGA002049	84.054868	2.1099607	1.139E-05
BGIBMGA002077	17.763987	2.746572	0.0096779
BGIBMGA002216	53.722536	2.7341956	8.096E-06
BGIBMGA002365	22.136267	2.0225709	0.0350008
BGIBMGA002367	32.333466	2.3387257	0.0024566
BGIBMGA002505	17.405611	2.4345519	0.0232845
BGIBMGA002576	22.705304	2.5157456	0.0085141
BGIBMGA002584	41.543012	4.7049828	6.882E-06
BGIBMGA002596	1118.8352	2.0294585	5.251E-44
BGIBMGA002902	46.449271	2.1190104	0.0007436
BGIBMGA002986	61.731213	4.9364762	6.233E-12
BGIBMGA002989	336.99884	2.3304161	5.648E-22
BGIBMGA003149	23.991594	3.2457697	0.0007062
BGIBMGA003217	17.340581	2.5195977	0.0181917
BGIBMGA003268	184.51383	2.0460034	2.491E-08
BGIBMGA003269	94.800387	2.0536451	2.774E-05
BGIBMGA003503	14.692013	2.5337952	0.0381396
BGIBMGA003514	21.750005	2.5033792	0.0074963
BGIBMGA003568	14.247744	4.7198095	0.0017829
BGIBMGA003660	19.112572	3.3103772	0.0022844
BGIBMGA003714	21.867749	2.0267961	0.0406657
BGIBMGA003740	21.830783	2.9300571	0.0024897
BGIBMGA003754	13.438252	2.4425187	0.0443467
BGIBMGA003783	1178.0396	5.4245354	4.27E-181
BGIBMGA003817	14.564689	2.3430032	0.048487

ID	AveExpr	Foldchange	P.value
BGIBMGA003901	40.626735	3.8497011	8.444E-07
BGIBMGA003917	68.94662	2.0919563	8.14E-05
BGIBMGA003959	21.345082	2.0845722	0.0312348
BGIBMGA003960	27.005709	2.1031489	0.0120503
BGIBMGA004060	23.788104	5.5024796	1.181E-05
BGIBMGA004172	30.575344	2.0196537	0.0118641
BGIBMGA004242	9.6882242	5.1264547	0.036459
BGIBMGA004243	226.47942	2.0838435	1.077E-12
BGIBMGA004280	75.872496	2.3172612	3.533E-06
BGIBMGA004300	37.496932	2.085937	0.0038466
BGIBMGA004353	20.815067	3.350239	0.0091095
BGIBMGA004391	14.504449	2.4079314	0.0489974
BGIBMGA004413	24.084009	8.8221992	3.134E-07
BGIBMGA004414	476.38309	4.3963989	3.608E-69
BGIBMGA004460	804.25814	2.1016784	2.933E-37
BGIBMGA004497	21.504582	2.9209617	0.0038615
BGIBMGA004598	302.04485	2.7846965	2.3E-15
BGIBMGA004614	3118.4549	2.1177469	2.028E-97
BGIBMGA004653	3.3497192	Inf	0.0334813
BGIBMGA004707	14.670973	4.0015383	0.0033332
BGIBMGA004730	40.84427	2.4745998	0.0007204
BGIBMGA004810	4.2449561	13.533281	0.0334884
BGIBMGA004911	845.68059	2.6527586	1.608E-10
BGIBMGA004945	262.77131	2.5399626	1.864E-10
BGIBMGA005012	56.503043	2.1971077	0.000141
BGIBMGA005073	16.759908	2.598175	0.0172961
BGIBMGA005135	69.596435	2.3522867	5.293E-05
BGIBMGA005143	29.800261	2.1449161	0.0065811
BGIBMGA005190	64.248168	2.0677736	0.0001451
BGIBMGA005302	1394.9316	2.3430063	3.916E-18
BGIBMGA005303	4831.6454	2.353802	3.757E-05
BGIBMGA005330	38.067876	2.0011933	0.0291181
BGIBMGA005572	49.331596	2.1862336	0.0004283
BGIBMGA005602	489.08567	2.2327122	6.671E-23
BGIBMGA005664	5.4756204	5.5968658	0.0489146
BGIBMGA005780	35.382342	2.103128	0.0061942
BGIBMGA005785	22.804918	3.4718007	0.0024049
BGIBMGA005788	15.02506	2.397927	0.0436682
BGIBMGA005860	1389.7075	2.2020372	1.103E-36
BGIBMGA005861	3515.4268	2.0652036	5.939E-60
BGIBMGA006032	11.406999	3.5030032	0.0308182
BGIBMGA006112	24.829149	2.0641969	0.0209085
BGIBMGA006125	24.400953	2.2746661	0.0128961

ID	AveExpr	Foldchange	P.value
BGIBMGA006280	37.762893	2.6204853	0.0002068
BGIBMGA006337	5.8249174	Inf	0.0017629
BGIBMGA006338	17.160365	2.3176859	0.0349791
BGIBMGA006414	17775.11	2.1390059	2E-188
BGIBMGA006457	26.226337	2.9174471	0.0009611
BGIBMGA006553	18.934589	4.6450224	0.0001954
BGIBMGA006671	130.53596	2.1870999	1.449E-06
BGIBMGA006882	101.71034	2.1139487	1.973E-05
BGIBMGA006949	10.928144	6.4092574	0.0026798
BGIBMGA007144	326.9052	2.8586935	6.978E-30
BGIBMGA007147	77.996342	2.0309512	5.111E-05
BGIBMGA007316	340.14936	2.6493194	9.361E-28
BGIBMGA007354	26.716828	4.2947223	8.6E-05
BGIBMGA007421	25.522953	5.3277231	9.198E-06
BGIBMGA007552	19.017924	2.0269745	0.0450523
BGIBMGA007684	242.51355	2.3182842	2.555E-08
BGIBMGA007697	1036.9321	2.0853912	1.18E-44
BGIBMGA007699	45.267033	2.066829	0.0016172
BGIBMGA007701	12368.665	2.1064721	4.56E-168
BGIBMGA007834	30.880829	2.0304585	0.0123313
BGIBMGA008050	33.540857	2.2902526	0.0028449
BGIBMGA008068	9.1862733	3.801849	0.0329067
BGIBMGA008115	10.872695	3.1440437	0.0246884
BGIBMGA008127	32.816933	2.7076247	0.0004727
BGIBMGA008132	312.29014	3.7061315	8.727E-37
BGIBMGA008177	5.6839	6.0699445	0.0373373
BGIBMGA008320	24.220412	2.0193127	0.0245121
BGIBMGA008398	9.4453881	5.0771914	0.0082814
BGIBMGA008401	355.50441	2.7653793	7.926E-31
BGIBMGA008418	19.57071	2.1114031	0.0330896
BGIBMGA008431	10.939781	5.3859543	0.0046395
BGIBMGA008594	34.848039	2.0953396	0.0037575
BGIBMGA008632	9.4755077	4.6262248	0.0387599
BGIBMGA008655	36.585268	2.5542061	0.0004959
BGIBMGA008656	8.8438226	3.47898	0.0413779
BGIBMGA008803	45.146731	2.2257789	0.0005514
BGIBMGA008855	17.831072	2.1838208	0.0327379
BGIBMGA008903	5718.2016	2.7057137	1.374E-09
BGIBMGA008979	6.0817995	5.0340509	0.0479278
BGIBMGA008987	22.126863	2.2425348	0.013614
BGIBMGA009037	360.99255	2.0231151	1.448E-13
BGIBMGA009120	101.37679	2.6927796	1.077E-09
BGIBMGA009123	11.34914	4.0090892	0.0109842

ID	AveExpr	Foldchange	P.value
BGIBMGA009148	368.43178	2.1440586	1.726E-20
BGIBMGA009436	14.937259	3.1291749	0.0147979
BGIBMGA009456	12.979937	3.320889	0.0156441
BGIBMGA009490	27.163153	2.6030188	0.0025475
BGIBMGA009551	60.234411	3.0792138	1.375E-07
BGIBMGA009584	36.673394	2.0716355	0.0175803
BGIBMGA009614	77.959199	3.1487267	1.352E-09
BGIBMGA009675	12.367088	2.9204268	0.0271743
BGIBMGA009776	10.155442	3.6802755	0.023519
BGIBMGA009799	218.78498	2.0772999	2.051E-12
BGIBMGA009812	23.501103	2.7092866	0.0046047
BGIBMGA009828	25.233895	2.985439	0.0013222
BGIBMGA009833	18.488086	2.0558949	0.0377775
BGIBMGA009857	73.471082	2.5694069	3.916E-07
BGIBMGA009960	25.280117	2.1216847	0.0134339
BGIBMGA010104	115.99011	2.2310844	2.836E-08
BGIBMGA010403	387.17196	2.3152472	6.552E-25
BGIBMGA010461	127.88789	2.3882457	1.008E-06
BGIBMGA010530	3.816937	12.16872	0.0489939
BGIBMGA010701	42.107258	2.3225407	0.0032199
BGIBMGA010825	57.837935	3.7134376	1.55E-08
BGIBMGA011048	43.323905	2.0615648	0.0022427
BGIBMGA011090	191.59617	2.1309133	8.6E-09
BGIBMGA011126	20.089088	2.1694835	0.0280056
BGIBMGA011170	109.00623	2.1608295	2.51E-07
BGIBMGA011253	1003.3577	2.0820039	1.997E-07
BGIBMGA011346	34.637527	2.9192198	0.0001538
BGIBMGA011573	37.033826	2.2964652	0.0012895
BGIBMGA011607	69.008915	2.6714427	6.004E-07
BGIBMGA011620	36653.875	2.0156831	4.31E-180
BGIBMGA011629	46.638891	2.002543	0.0021877
BGIBMGA011685	4.0483131	14.245793	0.0464261
BGIBMGA011698	22.450831	2.0472624	0.0261284
BGIBMGA011787	71.162555	3.6355852	2.807E-10
BGIBMGA011811	20.200163	2.576947	0.0265532
BGIBMGA011832	41.950167	2.1021593	0.008749
BGIBMGA011948	12939.476	2.0534375	1.42E-160
BGIBMGA012157	16.672107	2.2141196	0.0415522
BGIBMGA012236	51.952779	2.2458135	0.0002827
BGIBMGA012340	7.8467671	4.285825	0.0315478
BGIBMGA012362	92.431826	2.5524525	1.323E-05
BGIBMGA012363	34.440884	3.1406199	7.535E-05
BGIBMGA012488	133.53465	3.9135325	4.23E-14

ID	AveExpr	Foldchange	P.value
BGIBMGA012626	22445.623	2.0351194	6.37E-169
BGIBMGA012646	40.702899	2.5381513	0.0002572
BGIBMGA012668	16.850267	2.4922463	0.0221964
BGIBMGA012676	66.38124	2.0916799	0.0001147
BGIBMGA012786	10.067641	6.6701572	0.0298737
BGIBMGA012836	21.553185	2.9746766	0.0191958
BGIBMGA012943	4.0923021	12.548751	0.0497241
BGIBMGA012999	4.2264731	Inf	0.0101871
BGIBMGA013111	1343.8065	2.3652635	2.367E-71
BGIBMGA013130	9.850134	6.1824997	0.0034257
BGIBMGA013138	90.967376	2.2642274	2.009E-06
BGIBMGA013379	52.790009	3.0994932	7.939E-07
BGIBMGA013511	951.4603	2.1264365	2.201E-27
BGIBMGA013604	9.3829162	4.3659015	0.0170302
BGIBMGA013748	10.812456	2.5961565	0.0461292
BGIBMGA013775	23.725484	2.0087399	0.0270114
BGIBMGA013781	95.956823	2.3115884	9.337E-07
BGIBMGA013844	6.5283016	22.972737	0.0034802
BGIBMGA013849	27.28792	2.3049014	0.0069295
BGIBMGA013865	6.61387	11.06287	0.009207
BGIBMGA013882	14.921009	2.4412287	0.0344361
BGIBMGA013931	63.824585	2.0066893	0.0003048
BGIBMGA013966	252.4164	2.424399	4.8E-16
BGIBMGA013975	23.820132	2.9831506	0.0479644
BGIBMGA013991	20.843131	2.1982246	0.0248487
BGIBMGA014204	13.479832	2.9336555	0.0234071
BGIBMGA014329	11.349317	3.3388155	0.0195427
BGIBMGA014372	32.613119	2.3312143	0.0017736
BGIBMGA014391	8.4413096	4.5989954	0.0187973
BGIBMGA014404	214.94509	2.5277705	3.012E-17
BGIBMGA014407	28.317505	2.1894101	0.0084151
BGIBMGA014460	14.363432	6.9703052	0.00033
BGIBMGA014465	15.38138	2.4994012	0.0239856
BGIBMGA014547	87.880884	2.1592844	2.924E-06
BGIBMGA014617	29.726152	2.6583324	0.0012853

Down

ID	AveExpr	Foldchange	P.value
BGIBMGA000315	5.2928469	0.2863517	0.0026305
BGIBMGA000444	3787.287	0.3573263	6.17E-288
BGIBMGA000520	8.6056002	0.1925695	4.594E-09
BGIBMGA000659	1.3232559	0.1403396	0.017968
BGIBMGA000670	1.3903414	0.1922553	0.0284808

ID	AveExpr	Foldchange	P.value
BGIBMGA000863	166.24518	0.4766407	2.928E-18
BGIBMGA000864	877.90238	0.4471482	1.754E-82
BGIBMGA000941	36.712593	0.4536212	5.055E-06
BGIBMGA001092	33.126707	0.3535861	1.185E-09
BGIBMGA001146	19.552227	0.1527634	5.496E-27
BGIBMGA001198	0.795799	0.1024946	0.0047481
BGIBMGA001466	0.330814	0.0763017	0.0449344
BGIBMGA001575	195.60373	0.3643825	3.16E-42
BGIBMGA001576	575.27781	0.397187	8.681E-84
BGIBMGA001611	20.32252	0.4046733	5.01E-05
BGIBMGA001890	8.1960641	0.4572919	0.0370473
BGIBMGA002204	2.6880913	0.2934504	0.0318005
BGIBMGA002389	9.3596429	0.4185649	0.0091531
BGIBMGA002436	63.667643	0.4058019	0.0070336
BGIBMGA002437	45.044884	0.3368735	3.563E-13
BGIBMGA002474	2.1375378	0.1363687	0.0004385
BGIBMGA002475	6.6693189	0.2229814	5.848E-06
BGIBMGA002548	0	0	0.0420155
BGIBMGA002847	5.3229665	0.3864999	0.0364579
BGIBMGA002937	1.0109249	0.0696487	2.32E-05
BGIBMGA003045	482.99241	0.404044	6.633E-69
BGIBMGA003122	16.16331	0.411832	0.0005035
BGIBMGA003151	1910.29	0.4943686	1.464E-96
BGIBMGA003167	17.43094	0.2238009	2.912E-13
BGIBMGA003170	20.88233	0.2051721	9.413E-18
BGIBMGA003171	157.71748	0.1551452	5.18E-116
BGIBMGA003203	144.75441	0.4462013	3.386E-11
BGIBMGA003249	12.258247	0.4511355	0.0113775
BGIBMGA003497	1359.8267	0.4379244	1.319E-41
BGIBMGA003572	0	0	0.0018425
BGIBMGA003705	72.749688	0.4751996	2.023E-09
BGIBMGA004037	337.12769	0.4767288	2.181E-33
BGIBMGA004303	40.205385	0.4794905	1.777E-05
BGIBMGA004470	0.330814	0.051383	0.0058015
BGIBMGA005013	51.781642	0.3893134	2.191E-11
BGIBMGA005060	28.965263	0.4542471	6.181E-05
BGIBMGA005891	14.696479	0.4095929	0.0018978
BGIBMGA006318	14.516086	0.4966203	0.0118912
BGIBMGA006351	103.45444	0.4513327	3.286E-14
BGIBMGA006413	6.3686245	0.3688452	0.0105038
BGIBMGA006678	11.226606	0.2924224	1.465E-05
BGIBMGA006699	60.645826	0.4195153	1.468E-06
BGIBMGA006875	327.4561	0.3479214	1.831E-50

ID	AveExpr	Foldchange	P.value
BGIBMGA006886	392.09756	0.4209919	1.992E-22
BGIBMGA006894	53.150766	0.4749398	4.39E-07
BGIBMGA007045	4.5271675	0.3505699	0.0325324
BGIBMGA007199	15.545494	0.3929363	0.0002507
BGIBMGA007223	17.659935	0.449025	0.0019189
BGIBMGA007297	1148.8668	0.4760931	3.953E-81
BGIBMGA007630	161.32491	0.4886682	2.337E-12
BGIBMGA007740	1257.8935	0.4724193	2.714E-88
BGIBMGA008361	1.3602218	0.1892682	0.0341131
BGIBMGA008374	2.2046233	0.1419718	0.0001764
BGIBMGA008607	1.4273073	0.2021414	0.0345426
BGIBMGA008856	44.258341	0.4338125	1.241E-07
BGIBMGA008986	8.9432605	0.422817	0.0161563
BGIBMGA009184	19.05489	0.4307932	0.0004428
BGIBMGA009479	814.87172	0.4530306	2.974E-75
BGIBMGA010112	0	0	0.0198057
BGIBMGA010221	411.96679	0.4925487	1.079E-22
BGIBMGA010535	0	0	0.027602
BGIBMGA010561	460.69676	0.4935156	3.565E-31
BGIBMGA010652	17.375314	0.397328	0.0001034
BGIBMGA010860	1.1936985	0.1720094	0.0187756
BGIBMGA010987	2.8361317	0.3085091	0.048932
BGIBMGA011010	12.746505	0.3871869	0.001112
BGIBMGA011227	4.9736695	0.32119	0.0096426
BGIBMGA011261	0.330814	0.0746435	0.0435301
BGIBMGA011304	20.972512	0.145424	1.392E-31
BGIBMGA011441	73.313638	0.2304877	2.537E-45
BGIBMGA011525	1409.407	0.4848946	6.147E-87
BGIBMGA011821	194.34909	0.4910325	3.492E-14
BGIBMGA011876	1.0294078	0.1610548	0.0316077
BGIBMGA011934	19.135845	0.4997924	0.0064284
BGIBMGA011942	7.9509953	0.2169225	7.98E-05
BGIBMGA012345	9.8129913	0.2864069	2.317E-05
BGIBMGA012346	57.798412	0.3204995	6.943E-16
BGIBMGA012439	8.813703	0.4741638	0.0388633
BGIBMGA012511	13.433462	0.1399982	1.211E-21
BGIBMGA012883	22.864656	0.2301594	2.87E-08
BGIBMGA013003	0	0	0.0260678
BGIBMGA013007	36.104181	0.4113289	1.242E-07
BGIBMGA013008	4.0923021	0.2752576	0.0077173
BGIBMGA013909	267.49429	0.4681508	3.436E-29

Table S4. Transcriptome of control vs. the knock down of *Mondo* in BmNs cells.

Up

ID	AveExpr	Foldchange	P.value
BGIBMGA000030	71.185363	2.0824685	5.681E-05
BGIBMGA000116	24.547058	3.9991812	0.0001115
BGIBMGA000117	194.60164	2.5257637	1.332E-15
BGIBMGA000223	21.594461	3.0924384	0.0014531
BGIBMGA000389	14.099644	2.4272366	0.0418729
BGIBMGA000476	542.44226	2.4751899	6.647E-37
BGIBMGA000477	20.221658	2.0553011	0.0392541
BGIBMGA000561	16.40784	3.1472723	0.0407726
BGIBMGA000590	488.23975	2.4437521	3.095E-33
BGIBMGA000736	22.40439	2.9547924	0.0015077
BGIBMGA000776	4.4025165	Inf	0.0162378
BGIBMGA001051	39.882192	2.4737447	0.0003385
BGIBMGA001063	28.126313	2.2006033	0.0090703
BGIBMGA001121	15.752412	3.2276661	0.0064595
BGIBMGA001161	8.9302088	5.7007207	0.0268313
BGIBMGA001193	12.533184	3.5812494	0.0098971
BGIBMGA001274	20.391883	2.730966	0.0060666
BGIBMGA001367	232.52977	2.8376519	7.606E-22
BGIBMGA001376	22.114679	5.8584301	0.033853
BGIBMGA001384	5.9230201	9.1735476	0.0139565
BGIBMGA001513	45.116606	2.0707309	0.0013615
BGIBMGA001546	2971.7963	2.0148475	1.506E-78
BGIBMGA001613	632.88124	2.127252	1.962E-31
BGIBMGA001642	68.945093	2.0014333	0.000149
BGIBMGA001721	26.114713	2.1158406	0.0133526
BGIBMGA001836	24.87537	2.5125757	0.0043943
BGIBMGA001993	54.701602	2.3510713	7.094E-05
BGIBMGA002015	11.931153	2.8232228	0.0354935
BGIBMGA002049	81.852545	2.0333637	3.312E-05
BGIBMGA002077	16.145062	2.4711017	0.0227147
BGIBMGA002216	69.172857	3.4834735	2.657E-08
BGIBMGA002285	4.0006749	12.637544	0.0466712
BGIBMGA002367	34.479389	2.4684973	0.0007395
BGIBMGA002438	29.463547	2.0260829	0.0128145
BGIBMGA002505	19.84739	2.7487167	0.0056051
BGIBMGA002526	39.893774	2.240954	0.0012913
BGIBMGA002584	32.209154	3.6101773	2.357E-05
BGIBMGA002986	62.723726	4.9646753	4.325E-12
BGIBMGA002989	329.94871	2.258436	6.688E-10
BGIBMGA003149	18.261352	2.4456365	0.0188304
BGIBMGA003217	15.922636	2.2912743	0.0344279

ID	AveExpr	Foldchange	P.value
BGIBMGA003286	55.566123	2.2007406	0.000635
BGIBMGA003299	3.9431361	12.455787	0.0473812
BGIBMGA003514	27.912524	3.1810645	0.0002306
BGIBMGA003552	66.383951	2.2887628	2.1E-05
BGIBMGA003660	17.715664	3.0366384	0.0057838
BGIBMGA003740	17.90252	2.3791827	0.0191072
BGIBMGA003783	1170.8259	5.3361635	2.61E-174
BGIBMGA003792	28.696629	2.0855465	0.0119879
BGIBMGA003817	19.42597	3.0935112	0.0027437
BGIBMGA003901	44.144449	4.1420388	7.365E-08
BGIBMGA003904	13.961135	3.6862355	0.0056332
BGIBMGA003917	67.566952	2.0295138	0.0001135
BGIBMGA003960	30.605641	2.3606111	0.0171291
BGIBMGA004060	17.304631	3.9629556	0.0011584
BGIBMGA004172	32.984068	2.1569794	0.0040861
BGIBMGA004243	261.81233	2.3845098	1.51E-18
BGIBMGA004340	20.285996	2.0877572	0.0320333
BGIBMGA004353	19.791047	3.1540942	0.0022094
BGIBMGA004413	32.259252	11.69364	1.152E-09
BGIBMGA004414	485.18643	4.4324475	1.286E-33
BGIBMGA004497	16.92651	2.2756672	0.0498127
BGIBMGA004598	251.6414	2.2963863	4.646E-15
BGIBMGA004614	3178.59	2.1365578	8.523E-60
BGIBMGA004773	13.445411	2.470078	0.0473327
BGIBMGA004789	131.53058	2.2040593	5.287E-09
BGIBMGA004790	3.1394529	Inf	0.0359899
BGIBMGA004802	16.295709	3.0696481	0.0070701
BGIBMGA004945	250.43227	2.3956496	6.764E-18
BGIBMGA005012	55.258585	2.1274174	0.0001871
BGIBMGA005156	5.2607923	8.3090345	0.0270952
BGIBMGA005190	79.732402	2.5396893	9.478E-06
BGIBMGA005341	20.022664	2.1827958	0.0265623
BGIBMGA005417	19.156393	3.280855	0.0024428
BGIBMGA005619	21.191424	2.0511666	0.0364263
BGIBMGA005633	18.062358	2.1524382	0.0375103
BGIBMGA005642	147.49181	2.0503636	1.22E-08
BGIBMGA005709	23.286386	2.8278329	0.0025005
BGIBMGA005745	6.718708	4.288979	0.0457313
BGIBMGA005763	43.243163	2.1169377	0.0326663
BGIBMGA005780	37.54228	2.2087884	0.0018041
BGIBMGA005785	16.453796	2.4807166	0.0187672
BGIBMGA005795	12.082995	2.812395	0.0349714
BGIBMGA006032	11.428763	3.4709275	0.0205039

ID	AveExpr	Foldchange	P.value
BGIBMGA006061	11.12922	3.8437637	0.0307136
BGIBMGA006086	9.5310446	4.4577561	0.0138827
BGIBMGA006110	11.23391	4.1094804	0.0270683
BGIBMGA006112	26.376295	2.1708742	0.0092941
BGIBMGA006125	24.524822	2.2627339	0.0392286
BGIBMGA006266	33.66882	2.1403538	0.0177134
BGIBMGA006280	34.080494	2.3407713	0.0016435
BGIBMGA006320	19.84739	2.0663617	0.0377429
BGIBMGA006337	4.9992102	Inf	0.004222
BGIBMGA006338	18.453813	2.467274	0.0158018
BGIBMGA006420	3.1866047	Inf	0.0353323
BGIBMGA006457	24.856987	2.7379654	0.0019694
BGIBMGA006553	16.306096	3.9611923	0.0015603
BGIBMGA006671	137.06839	2.2724236	1.198E-09
BGIBMGA006854	28.845525	2.008031	0.0204705
BGIBMGA006882	107.14814	2.2036007	3.788E-06
BGIBMGA006949	10.867083	6.2953565	0.0031644
BGIBMGA006950	19.996841	2.2247882	0.0249315
BGIBMGA007029	41.471817	2.0766921	0.0023683
BGIBMGA007101	4.128797	14.350998	0.0348649
BGIBMGA007144	321.89846	2.7862033	3.787E-20
BGIBMGA007316	343.75512	2.6503645	3.422E-28
BGIBMGA007354	15.38109	2.447888	0.0302669
BGIBMGA007403	16.608585	2.7634821	0.0438455
BGIBMGA007421	28.022818	5.7864086	1.144E-06
BGIBMGA007561	28.870707	2.0518285	0.0102621
BGIBMGA007654	14.971253	2.2883973	0.0463816
BGIBMGA007697	1009.8395	2.0103087	2.515E-39
BGIBMGA007713	13.763336	4.0280357	0.0038247
BGIBMGA007883	19.800238	2.4596672	0.0125961
BGIBMGA007991	10.829123	3.0384193	0.0381032
BGIBMGA008050	37.465163	2.5319212	0.0171637
BGIBMGA008067	43.625138	2.0460171	0.0099358
BGIBMGA008068	10.296767	4.2163058	0.0129076
BGIBMGA008080	21.51854	2.0958928	0.0255397
BGIBMGA008102	7.6792827	3.4325239	0.0495641
BGIBMGA008127	31.446377	2.5688214	0.0009596
BGIBMGA008132	343.07194	4.0299623	1.158E-15
BGIBMGA008299	7.4384747	4.760078	0.0264511
BGIBMGA008352	4.5869816	15.94357	0.0235094
BGIBMGA008398	9.7834356	5.2060326	0.0329255
BGIBMGA008401	389.43963	2.9986189	1.297E-37
BGIBMGA008431	10.458997	5.0887968	0.0178672

ID	AveExpr	Foldchange	P.value
BGIBMGA008562	5.6626339	17.887427	0.0076387
BGIBMGA008628	6.3536311	6.8072382	0.0205632
BGIBMGA008649	10.43408	3.8398574	0.0481368
BGIBMGA008655	31.081301	2.1477057	0.0069779
BGIBMGA008656	9.4643146	3.6879163	0.0214209
BGIBMGA008793	13.943948	2.7959379	0.0223428
BGIBMGA008803	44.070919	2.149877	0.0012201
BGIBMGA008903	4295.5033	2.0121052	2.125E-08
BGIBMGA009037	408.88299	2.2680138	7.211E-19
BGIBMGA009120	95.877729	2.5207151	1.223E-08
BGIBMGA009179	88.206731	2.1083123	4.696E-05
BGIBMGA009215	14.971253	3.0994156	0.0121195
BGIBMGA009327	12.702213	3.3427837	0.0395602
BGIBMGA009455	17.587542	2.985133	0.006983
BGIBMGA009490	24.647606	2.3379982	0.007166
BGIBMGA009551	53.937982	2.7298543	3.067E-05
BGIBMGA009552	16.503894	2.2948425	0.0361509
BGIBMGA009577	360.87079	2.0299676	1.19E-17
BGIBMGA009614	67.567241	2.7017784	1.716E-05
BGIBMGA009642	3.7832984	Inf	0.0159035
BGIBMGA009672	9.5402358	3.102242	0.0451311
BGIBMGA009675	13.901845	3.2481035	0.0099005
BGIBMGA009812	23.711948	2.7041511	0.0464241
BGIBMGA009828	27.43412	3.2110616	0.0013743
BGIBMGA009829	41.892041	2.7790287	3.948E-05
BGIBMGA009833	25.666915	2.8256623	0.0011071
BGIBMGA009857	68.985711	2.387402	4.75E-06
BGIBMGA010104	115.25509	2.1943263	5.653E-08
BGIBMGA010167	14.332457	2.2619664	0.0453675
BGIBMGA010287	64.102774	2.233964	3.516E-05
BGIBMGA010400	9.0141254	4.1051287	0.0288298
BGIBMGA010403	345.02974	2.0424951	1.003E-17
BGIBMGA010461	116.79727	2.1582572	7.18E-05
BGIBMGA010500	18.521739	2.6220894	0.0122403
BGIBMGA010568	27.622172	2.5341105	0.0021882
BGIBMGA010701	58.138847	3.1727089	8.315E-07
BGIBMGA010769	5.5395609	6.4181915	0.0393591
BGIBMGA010825	63.524463	4.0343634	3.473E-10
BGIBMGA010983	10.66304	4.8201759	0.0060585
BGIBMGA011106	47.263682	3.0301363	0.0146287
BGIBMGA011170	143.44095	2.8137897	1.026E-10
BGIBMGA011216	17.278253	2.2375599	0.0343699
BGIBMGA011228	37.104869	2.403767	0.0005592

ID	AveExpr	Foldchange	P.value
BGIBMGA011313	20.640132	3.6610588	0.0020341
BGIBMGA011346	33.695839	2.8107175	0.0002646
BGIBMGA011348	16.977515	3.7067099	0.0022319
BGIBMGA011569	46.80109	2.0075205	0.0018726
BGIBMGA011573	33.699981	2.0691699	0.0067453
BGIBMGA011580	8.5692741	4.6946266	0.0189898
BGIBMGA011582	15.340183	2.4724462	0.0257333
BGIBMGA011679	7.8690857	3.5836655	0.0488131
BGIBMGA011787	67.184688	3.397165	2.792E-09
BGIBMGA011811	22.264685	2.8104998	0.0054007
BGIBMGA011831	29.358857	2.0365239	0.0106143
BGIBMGA011832	41.996732	2.0833102	0.002023
BGIBMGA011951	8.688205	5.4034601	0.0086343
BGIBMGA011981	6.841781	7.1103119	0.0145268
BGIBMGA012043	16.058754	2.6019731	0.0290434
BGIBMGA012216	16.774667	3.0563123	0.00788
BGIBMGA012236	60.897787	2.6041411	9.421E-06
BGIBMGA012326	13.209652	3.8989347	0.0050371
BGIBMGA012338	533.93256	2.1078347	1.113E-23
BGIBMGA012362	98.337127	2.6878392	1.213E-09
BGIBMGA012363	35.220485	3.1787383	4.724E-05
BGIBMGA012368	12.380146	3.5144179	0.0150078
BGIBMGA012456	372.35266	2.1744302	2.431E-21
BGIBMGA012488	163.6084	4.7450836	1.346E-25
BGIBMGA012521	74.90727	2.0075331	0.0006485
BGIBMGA012646	36.676009	2.2630063	0.001919
BGIBMGA012654	6.4479347	11.205964	0.0096803
BGIBMGA012676	68.606394	2.1395866	4.923E-05
BGIBMGA012736	4.7767846	15.08916	0.0407196
BGIBMGA012783	23.663311	2.0277075	0.0420892
BGIBMGA012836	23.510562	3.2111583	0.0009782
BGIBMGA012973	10.538771	3.0662174	0.0323204
BGIBMGA013101	13.958188	2.5583958	0.0328529
BGIBMGA013111	1174.6034	2.0464852	4.259E-28
BGIBMGA013130	8.0155901	4.9851403	0.0152735
BGIBMGA013211	8.6635777	3.52396	0.0438892
BGIBMGA013220	38.789353	2.2112881	0.0013578
BGIBMGA013274	13.85764	2.9289803	0.0228564
BGIBMGA013379	55.248487	3.2101823	2.826E-07
BGIBMGA013455	11.241351	2.8335417	0.0397613
BGIBMGA013479	12.332994	2.9661931	0.0206244
BGIBMGA013662	38.014439	2.0844656	0.0041005
BGIBMGA013748	16.901593	4.0212493	0.0009482

ID	AveExpr	Foldchange	P.value
BGIBMGA013839	466.47856	2.0927054	3.48E-23
BGIBMGA013844	6.1742152	21.460525	0.0094593
BGIBMGA013849	24.767733	2.0701293	0.0176752
BGIBMGA013865	6.4387435	10.655379	0.0096332
BGIBMGA013931	77.785429	2.4204653	0.0032954
BGIBMGA013932	3.944332	11.985486	0.04721
BGIBMGA013955	6.6899386	5.4063916	0.0278191
BGIBMGA013966	280.32729	2.6653735	1.248E-13
BGIBMGA013991	22.175719	2.3148054	0.0140665
BGIBMGA014055	19.433966	2.2189355	0.023846
BGIBMGA014204	14.056634	3.0282876	0.0163752
BGIBMGA014239	15.751216	2.4465082	0.0275245
BGIBMGA014263	4.9716367	7.5535581	0.0369587
BGIBMGA014309	16.687163	2.9777182	0.0198446
BGIBMGA014329	13.373632	3.8953462	0.0120007
BGIBMGA014364	17.229906	2.3598257	0.0275336
BGIBMGA014372	32.956494	2.3326317	0.0017242
BGIBMGA014404	208.18133	2.4235775	1.051E-15
BGIBMGA014407	29.78413	2.2781621	0.0089768
BGIBMGA014465	14.718862	2.367527	0.0436996
BGIBMGA014478	39.525751	2.1857741	0.0012953
BGIBMGA014574	12.318754	4.4920383	0.0105785
BGIBMGA014575	7.9254287	24.082688	0.0007435
BGIBMGA014617	29.656008	2.6238844	0.0012387
BGIBMGA014621	4.9808278	7.7142843	0.0369852

Down

ID	AveExpr	Foldchange	P.value
BGIBMGA000310	32.915235	0.4410241	0.0013744
BGIBMGA000315	5.9425984	0.3182778	0.0042727
BGIBMGA000444	3929.536	0.3669869	6.92E-244
BGIBMGA000520	15.078601	0.3339764	1.03E-05
BGIBMGA000659	1.6999196	0.1785746	0.0194449
BGIBMGA000673	56.097837	0.441035	5.473E-06
BGIBMGA000818	8.0363642	0.4504763	0.0353728
BGIBMGA000864	938.72331	0.4732522	5.836E-70
BGIBMGA001092	29.388822	0.3104723	1.333E-11
BGIBMGA001146	23.473797	0.1815418	7.555E-25
BGIBMGA001198	1.4579157	0.1859297	0.0180389
BGIBMGA001575	203.49619	0.3752509	2.454E-41
BGIBMGA001576	536.1043	0.3663725	1.351E-92
BGIBMGA001611	12.398528	0.2443716	1.441E-08
BGIBMGA002163	100.11773	0.4402201	1.277E-06

ID	AveExpr	Foldchange	P.value
BGIBMGA002192	11.101646	0.4452351	0.0100363
BGIBMGA002436	53.866203	0.3399354	0.0020786
BGIBMGA002437	42.753263	0.3165279	2.107E-15
BGIBMGA002474	1.785032	0.112778	0.000193
BGIBMGA002475	7.8966592	0.2613655	2.551E-05
BGIBMGA002548	0	0	0.0406831
BGIBMGA002974	323.05331	0.4928764	2.119E-28
BGIBMGA003045	553.31191	0.458165	1.104E-53
BGIBMGA003122	16.012798	0.4038174	0.0004381
BGIBMGA003167	26.47299	0.3364483	2.343E-08
BGIBMGA003170	31.565308	0.3069728	1.251E-12
BGIBMGA003171	203.31978	0.1979763	2.59E-115
BGIBMGA003203	139.2615	0.4249233	5.129E-16
BGIBMGA003249	13.060202	0.4758562	0.0402581
BGIBMGA003497	1507.7193	0.4806326	3.73E-34
BGIBMGA003572	0.3363074	0.0542803	0.0066042
BGIBMGA003869	22.293454	0.4664666	0.0007425
BGIBMGA003898	5.2320229	0.3896385	0.0369535
BGIBMGA004037	349.0331	0.4885664	3.324E-31
BGIBMGA004797	0	0	0.0238553
BGIBMGA004998	6.288097	0.364085	0.0396446
BGIBMGA005013	49.105698	0.3654551	7.423E-13
BGIBMGA005481	11.373615	0.451095	0.0111741
BGIBMGA005710	3.0923011	0.301556	0.0349469
BGIBMGA005723	379.4768	0.4534904	9.831E-42
BGIBMGA005814	2.0270358	0.1419166	0.0005471
BGIBMGA005891	13.578871	0.3744896	0.0002525
BGIBMGA006015	0	0	0.0015769
BGIBMGA006111	2.09257	0.260195	0.0419818
BGIBMGA006194	1361.73	0.4852916	2.072E-80
BGIBMGA006318	11.752933	0.3978979	0.0060587
BGIBMGA006745	1.0089223	0.1468529	0.0471979
BGIBMGA006875	284.84521	0.2995999	1.614E-63
BGIBMGA006886	383.82397	0.4079719	6.361E-21
BGIBMGA006978	15.140282	0.485351	0.0070896
BGIBMGA007297	1200.8074	0.4925583	1.17E-71
BGIBMGA007651	7.4384747	0.4322084	0.0286377
BGIBMGA007740	1295.5173	0.4815989	4.736E-80
BGIBMGA007907	4.9520584	0.3321572	0.0129552
BGIBMGA008361	1.7562625	0.2419705	0.0493489
BGIBMGA008374	5.476973	0.3492423	0.0122896
BGIBMGA008502	9.2694626	0.4869966	0.0448013
BGIBMGA008856	48.487676	0.4704161	6.558E-07

ID	AveExpr	Foldchange	P.value
BGIBMGA009184	21.078738	0.4717672	0.0014323
BGIBMGA009424	8.2783681	0.41013	0.0359502
BGIBMGA009498	67.089743	0.4884122	3.242E-08
BGIBMGA009572	1242.5303	0.4939659	4.526E-73
BGIBMGA010029	1678.8701	0.4987019	1.522E-82
BGIBMGA010652	17.542782	0.3971019	0.000114
BGIBMGA011141	0	0	0.0085731
BGIBMGA011167	0	0	0.0165231
BGIBMGA011304	43.896552	0.3012532	2.095E-17
BGIBMGA011378	16.72632	0.4690234	0.0029889
BGIBMGA011441	100.81295	0.3137176	8.118E-33
BGIBMGA011525	1419.753	0.4835063	4.263E-75
BGIBMGA011530	22.163582	0.421105	7.73E-05
BGIBMGA011781	0	0	0.0473304
BGIBMGA011825	0	0	0.0475932
BGIBMGA011876	1.0181135	0.1576159	0.0275733
BGIBMGA011942	6.9986725	0.1889937	6.835E-06
BGIBMGA012345	11.509733	0.332569	0.0001365
BGIBMGA012346	69.554831	0.3817625	9.161E-13
BGIBMGA012511	16.300758	0.1681667	1.097E-19
BGIBMGA012883	40.140827	0.4000011	9.791E-07
BGIBMGA013007	33.292801	0.375466	1.332E-08
BGIBMGA013008	3.6931369	0.2459368	0.0023602
BGIBMGA013329	12.035843	0.4990553	0.0273541
BGIBMGA013372	3.5700639	0.3159489	0.0277468
BGIBMGA013759	407.03785	0.4787504	1.672E-37
BGIBMGA014528	8.6226708	0.4352372	0.0272507

Table S5. Genes regulated by both BmMlx and BmMondo in BmNs cells.

Down-regulated

ID
BGIBMGA000315
BGIBMGA000444
BGIBMGA000520
BGIBMGA000659
BGIBMGA000864
BGIBMGA001092
BGIBMGA001146
BGIBMGA001198
BGIBMGA001575
BGIBMGA001576
BGIBMGA001611
BGIBMGA002436

ID
BGIBMGA002437
BGIBMGA002474
BGIBMGA002475
BGIBMGA002548
BGIBMGA003045
BGIBMGA003122
BGIBMGA003167
BGIBMGA003170
BGIBMGA003171
BGIBMGA003203
BGIBMGA003249
BGIBMGA003497
BGIBMGA003572
BGIBMGA004037
BGIBMGA005013
BGIBMGA005891
BGIBMGA006318
BGIBMGA006875
BGIBMGA006886
BGIBMGA007297
BGIBMGA007740
BGIBMGA008361
BGIBMGA008374
BGIBMGA008856
BGIBMGA009184
BGIBMGA010652
BGIBMGA011304
BGIBMGA011441
BGIBMGA011525
BGIBMGA011876
BGIBMGA011942
BGIBMGA012345
BGIBMGA012346
BGIBMGA012511
BGIBMGA012883
BGIBMGA013007
BGIBMGA013008

Up-regulated

ID
BGIBMGA000116
BGIBMGA000117
BGIBMGA000223

ID
BGIBMGA000476
BGIBMGA000561
BGIBMGA000590
BGIBMGA000736
BGIBMGA001367
BGIBMGA001376
BGIBMGA001384
BGIBMGA001613
BGIBMGA001836
BGIBMGA002049
BGIBMGA002077
BGIBMGA002216
BGIBMGA002367
BGIBMGA002505
BGIBMGA002584
BGIBMGA002986
BGIBMGA002989
BGIBMGA003149
BGIBMGA003217
BGIBMGA003514
BGIBMGA003660
BGIBMGA003740
BGIBMGA003783
BGIBMGA003817
BGIBMGA003901
BGIBMGA003917
BGIBMGA003960
BGIBMGA004060
BGIBMGA004172
BGIBMGA004243
BGIBMGA004353
BGIBMGA004413
BGIBMGA004414
BGIBMGA004497
BGIBMGA004598
BGIBMGA004614
BGIBMGA004945
BGIBMGA005012
BGIBMGA005190
BGIBMGA005780
BGIBMGA005785
BGIBMGA006032
BGIBMGA006112

ID
BGIBMGA006125
BGIBMGA006280
BGIBMGA006337
BGIBMGA006338
BGIBMGA006457
BGIBMGA006553
BGIBMGA006671
BGIBMGA006882
BGIBMGA006949
BGIBMGA007144
BGIBMGA007316
BGIBMGA007354
BGIBMGA007421
BGIBMGA007697
BGIBMGA008050
BGIBMGA008068
BGIBMGA008127
BGIBMGA008132
BGIBMGA008398
BGIBMGA008401
BGIBMGA008431
BGIBMGA008655
BGIBMGA008656
BGIBMGA008803
BGIBMGA008903
BGIBMGA009037
BGIBMGA009120
BGIBMGA009490
BGIBMGA009551
BGIBMGA009614
BGIBMGA009675
BGIBMGA009812
BGIBMGA009828
BGIBMGA009833
BGIBMGA009857
BGIBMGA010104
BGIBMGA010403
BGIBMGA010461
BGIBMGA010701
BGIBMGA010825
BGIBMGA011170
BGIBMGA011346
BGIBMGA011573

ID
BGIBMGA011787
BGIBMGA011811
BGIBMGA011832
BGIBMGA012236
BGIBMGA012362
BGIBMGA012363
BGIBMGA012488
BGIBMGA012646
BGIBMGA012676
BGIBMGA012836
BGIBMGA013111
BGIBMGA013130
BGIBMGA013379
BGIBMGA013748
BGIBMGA013844
BGIBMGA013849
BGIBMGA013865
BGIBMGA013931
BGIBMGA013966
BGIBMGA013991
BGIBMGA014204
BGIBMGA014329
BGIBMGA014372
BGIBMGA014404
BGIBMGA014407
BGIBMGA014465
BGIBMGA014617

Table S6. Primers for q-PCR.

Primers name	Sequence（5'→ 3'）
β-actin-F	CTCCCTCGAGAAGTCCTACGAACT
β-actin-R	GGATGCCGCACGATTCCATACF
Mlx-F	CTCACACACAGGCAGAGCAG
Mlx-R	CTGAAGGACTGCAGCTTTGC
Mondo-F	GGCTATACACGGTACGAGTAAC
Mondo-R	GGGGGGTATAGCTGCACTATATTTA
Tret-F	GAAACCAACGCTCTCAGCG
Tret-R	CTGATCTGACGTAGTTGTTG
PFK-F	CATAGCTGGAATGGTTGGTTC
PFK-R	GCCAAATAACCACAGTTGC
PK-F	CGAACAAGGCGTTGATATG
PK-R	CGGATTCAGCTATAATCTCG
Cabut-F	GACACTTAAATTTAAAATGAAC

Primers name	Sequence (5’→ 3’)
Cabut-R	GTGACGGTACGCGAGCTTCTTC
ACC-F	GTTCTCGTTGGACGACAAC
ACC- R	GACTCTGCATAAACTTCTCC
FAS-F	GACAACGTCACCATATCCGGTCC
FAS-R	CTCAGCTTGGCCAGCTCTGATC
GS2-F	GATAACGGTATCATTGAAATCG
GS2-R	CAGAGCGAGGTATTCTGATAC
Cycle-F	GACGCAGGACTGTGCGAACCCG
Cycle-R	GTTTTCAGGAACGTAGTGATTG
FAMet5-F	TTGTATACGGACATAATAGCTGCC
FAMet5-R	CCTTTCTACGGATTTTGTGTTCAC

Table S7. Primers for cloning.

Primers name	Sequence (5’→ 3’)
Mlx-F	ATGTATCCTCGTTGTGGCAG
Mlx-R	TTAAAGTGTGTAGTTCTGATC
Mondo-F	ATGAACCAGGTAGAAAAAAG
Mondo-R	TTACGAGGGTTCTGTCTTG
shGFP-F (Interfered in cells)	TCGAGCGAAGGTTATGTACAGGAAAGTGTGTGCTGTCCACTTTCCTGTACATAACCTTCA
shGFP-R (Interfered in cells)	GATCTGAAGGTTATGTACAGGAAAGTGGACAGCACACACTTTCCTGTACATAACCTTCGC
shMlx-F (Interfered in cells)	TCGAGCGAAGAGATGCAATTAAGAAAGGTGTGCTGTCCCTTTCTTAATTGCATCTCTTCGA
shMlx-R (Interfered in cells)	GATCTGAAGAGATGCAATTAAGAAAGGGACAGCACACCTTTCTTAATTGCATCTCTTCGC
shMondo-F (Interfered in cells)	TCGAGCGGATCGACTAGCACAAGTAGCGTGTGCTGTCCGCTACTTGTGCTAGTCGATCCGA
shMondo-R (Interfered in cells)	GATCTGGATCGACTAGCACAAGTAGCGGACAGCACACGCTACTTGTGCTAGTCGATCCGC

The underline means intron.

Table S8. Primers for ChIP and EMSA probes.

Primers name	Sequence (5’→ 3’)
ACC-F (EMSA probe)	Biotin-AAGTACCCGCGTGATGATCGTACGATCGCAC
ACC-F (EMSA probe)	AAGTACCCGCGTGATGATCGTACGATCGCAC
ACC-R (EMSA probe)	GTGCGATCGTACGATCATCACGCGGGTACTT
ACC-F1 (Mutation probe)	AAGTACCAAAAAAATGATAAAAAAATCGCAC
ACC-R1 (Mutation probe)	GTGCGATTTTTTTATCATTTTTTTGGTACTT
Tret-F (ChIP)	CATCTACATTTTCAGGTGAGGTG
Tret-R (ChIP)	GGAAGGTTGGCAGTGTCGGC
PFK-F (ChIP)	CCCTACTTAAGCTAAAAGCC
PFK-R (ChIP)	GATCTTCTCAGTGGGTCGCG

Primers name	Sequence (5'→ 3')
ACC-F (ChIP)	GACGTACAAAAACCCAACC
ACC-R (ChIP)	CTGTTGCAAACCTCATTGC
FAS-F (ChIP)	GTAGGTATCTAACAGTGTAAG
FAS-R (ChIP)	GCACGATCATCGTTTTCGCCG
GS2-F (ChIP)	CACCGTGGAAGTCAATTGTG
GS2-R (ChIP)	GTCAACTCGAGTAACATCAG
Cabut-F (ChIP)	GCCTACTGAGTTTCTGGCGG
Cabut-R (ChIP)	CTAGAGAGACCATATCAGCG

Table S9. Primers for construction of transgenic vector.

Primers name	Sequence (5'→ 3')
Mlx-OE-F	GGATCCATGAACCAGGTAGAA AAAAG
Mlx-OE-R	GCGGCCGCTTACGAG GGTTCTGTCTTGACGG
Mondo-OE-F	GGATCCATGAACCAGGTAGAAAAAAG
Mondo-OE-R	GCGGCCGTTACGAGGGTTCTGTCTTG
Mlx-KO-F (Forward)	GGATCCGGCTATGATTCACTCCAAGAC
Mlx-KO-R (Forward)	GAATTCCTGAAACAGAACAAAGTCATTCGTCAGATAAC GGCCCACCGGCCGAGAGGCGAAGTGCATAGTCCAGAAT CGATGAGCTCACGCATTATACGAAGAGCGACAAC
Mlx-KO-F (Reverse)	GAATTCGCATTATACGAAGAGCGACAAC
Mlx-KO-R (Reverse)	CGGCCGCGGCTATGATTCACTCCAAGAC
Mondo-KO-F (Forward)	GGATCCCAACTTTCCATCGAAGTTTCC
Mondo-KO-R (Forward)	GAATTCCTGAAACAGAACAAAGTCATTCGTCAGATAACGGTCG ATAACGGCCCACCGGCCGAGAGGCGAAGTGCATAGTCCAGAAT CGATGAGCTCACATGAAGTCTGCTAAACTTGCCC
Mondo-KO-F (Reverse)	GAATTCATGAAGTCTGCTAAACTTGCC
Mondo-KO-R (Reverse)	GCGGCCGC CAACTTTCCATCGAAGTTTC
